# Cucurbitacin E Inhibits Proliferation and Migration of Intestinal Epithelial Cells via Activating Cofilin

**DOI:** 10.3389/fphys.2018.01090

**Published:** 2018-08-07

**Authors:** Huapei Song, Yu Wang, Li Li, Hehuan Sui, Pei Wang, Fengjun Wang

**Affiliations:** ^1^State Key Laboratory of Trauma, Burns, and Combined Injury, Institute of Burn Research, Southwest Hospital, Third Military Medical University (Army Medical University), Chongqing, China; ^2^Department of Gastroenterology, Southwest Hospital, Third Military Medical University (Army Medical University), Chongqing, China

**Keywords:** Cucurbitacin E, cofilin, intestinal epithelial cells, cell cycle, cell proliferation, cell migration, actin, LIM kinase

## Abstract

The proliferation and migration of intestinal epithelial cell is important to the barrier integrity of intestinal epithelium. Cucurbitacin E (CuE) is one of the tetracyclic triterpenoids extracted from the cucurbitaceae that has been shown to inhibit cancer cell growth, tumor angiogenesis and inflammatory response. Nevertheless, the role of Cucurbitacin E in regulating the proliferation and migration of intestinal epithelial cells remain unclear. In this study, the human intestinal epithelial cell line Caco-2 was treated with CuE and the effects of CuE on cell cycle, proliferation, migration and actin dynamics in Caco-2 cells were investigated successively. We found that CuE significantly inhibited the cell proliferation and migration, inducing the cell cycle arrest in G2/M phase and disrupting the actin dynamic balance in Caco-2 cells. Finally, we showed that CuE inhibited cofilin phosphorylation by suppressing the phosphorylation of both LIM kinase (LIMK)1 and LIMK2 *in vitro*, resulting in the activation of cofilin, which is closely associated with cell proliferation and migration. Therefore, our studies provided the first evidence that CuE inhibited the proliferation and migration of intestinal epithelial cells via activating cofilin, and CuE is a potential candidate in intestinal disease therapy.

## Introduction

Cell proliferation and migration plays an important role in intestinal epithelial repairment when it is damaged, such as by inflammatory bowel disease, ulcers, infections, radiation, or chemotherapy (Ciorba et al., 2012; Mokry et al., 2014; Zuo et al., 2015; Coch and Leube, 2016; Ramanan and Cadwell, 2016). It has been well recognized that the cytoskeleton plays a critical role during the processes of cell proliferation and migration. Actin, a key component of the cytoskeleton, is involved not only in the transportation of intracellular materials and muscle contraction but also in cell migration and cytokinesis (Mitsushima et al., 2010; Lancaster and Baum, 2014). Cofilin is a ubiquitous actin binding protein, containing an actin-depolymerizing factor homology domain, which enables stoichiometric binding of cofilin to both F- and G-actin (Pope et al., 2004). By severing the actin filaments to regulate the polymerization and depolymerization of actin, cofilin plays critical roles in the regulation of actin dynamics during cell development, migration, and tumor metastasis in a variety of cells (Samstag et al., 2013; Wu et al., 2016).

Cucurbitacin E is a family member of the tetracyclic triterpenoids extracted from the cucurbitaceae. The previous studies have shown that CuE disrupted cell actin and inhibited cell adhesion, and has inhibitory activity on cancer cell proliferation, actin polymerization and permeability (Dong et al., 2010). Nevertheless, the role of CuE in regulating the proliferation and migration of intestinal epithelial cells and its underlying mechanism remains unclear. In this study, we found that CuE inhibited Caco-2 proliferation and migration *in vitro*. To understand the molecular mechanism of CuE action, we found that CuE activated cofilin via inhibiting the phosphorylation of LIMK1 and LIMK2 in a dose-dependent manner.

## Materials and Methods

### Materials

The human intestinal epithelial cell line Caco-2 was purchased from ATCC. DMEM, nonessential amino acid (NEAA) and fetal bovine serum (FBS) were purchased from GIBCO (Thermo Fisher Scientific, MA, United States), and cell culture incubator and multi-plate reader were purchased from Thermo Fisher (Thermo Fisher Scientific, Waltham, MA, United States). Trypsin was purchased from BBI (BBI Life Sciences Corporation, Shanghai, China). Cell counting kit-8 (CCK-8) was purchased from the Beyotime Institute of Biotechnology (Shanghai, China). Inverted fluorescence microscope was purchased from Olympus (Japan). Twenty-four-well plate Transwell chambers (8 μm per well) were purchased from Corning (Corning, NY, United States). DU800 nucleic acid and protein analyzer was purchased from Beckman Coulter (Carlsbad, CA, United States). The Alexa Fluor 488- deoxyribonuclease and Alexa Fluor 594-phalloidin were purchased from Invitrogen (Thermo Fisher Scientific, Waltham, MA, United States). TCS SP5 laser confocal microscope was purchased from Leica (Germany). Anti-β-actin, anti-cofilin, and anti-phosphorylated cofilin were purchased from Sigma-Aldrich (St. Louis, MO, United States). Anti-LIM kinase 1 (LIMK1), LIMK2, anti-phosphorylated-LIMK1, anti-phosphorylated-LIMK2 antibody were purchased from Abcam (Cambridge, MA, United States). Hypersensitive ECL chemiluminescent substrate reagent was purchased from GE Healthcare (Chicago, IL, United States). Polyvinylidene fluoride (PVDF) membrane was purchased from Millipore (EMD Millipore, Billerica, MA, United States). UD-201 tissue and cell ultrasonicator were purchased from Tomy (Japan). A TGL-16G bench top centrifuge was purchased from Cence (Xiangyi Centrifuge, Inc., Hunan, China). RC DC protein assay kit, protein assay kit, protein electrophoresis reagents, electrophoresis cell, electroporation devices, and ChemiDoc XRS gel imaging system were purchased from Bio-Rad Laboratories (Hercules, CA, United States). Cucurbitacin E (CuE) was purchased from Chenguang Biotechnology Co., Ltd. (Baoji, Shaanxi, China). DMSO was formulated into 0.02 mol/L stock solution and preserved in -20°C for later use.

### Cell Culture

Caco-2 cells were grown in DMEM media (pH 7.4) containing 100 U/mL penicillin, 100 μg/mL streptomycin, 10% FBS, 2 mmol/L glutamine, and 1 mmol/L NEAA at 37°C in an incubator containing 5% CO_2_ and saturated humidity. Cell culture media were changed once every 2–3 days until 80% confluence, followed by partial digestion with Ca^2+^-free and Mg^2+^-free Hank’s balanced saline solution containing 0.25% trypsin and 0.53 mmol/L EDTA. The cells were subcultured in a ratio of 1:3.

### Cell Proliferation Assay

Caco-2 cells were counted after routine trypsinization, followed by seeding at 2 × 10^4^ cells/mL on a 96-well plate for adhesion overnight. The cells were treated with 0.001, 0.01, 0.1, 1, and 10 μmol/L CuE, with DMEM media as the blank and 0.05% DMSO (in DMEM) as the control. After incubation for 24, 48, and 72 h, the cell proliferation was assayed by CCK-8 kit in accordance with the manufacturer’s instructions.

### Flow Cytometry

With reference to the methods described in previous studies (Wu et al., 2010; Yang et al., 2010), Caco-2 cells were seeded overnight in six-well plates, followed by treatment with 0.001, 0.01, 0.1, 1, and 10 μmol/L CuE for 24 h, after which the cells were collected. Pre-chilled 70% ethanol was added to fix the cells at 4°C overnight. The cells were then washed twice with PBS and subjected to flow cytometry. ModFit3.2 software was used for data analysis.

### Cell Scratch Assay

The Caco-2 cells were seeded in 24-well plates to grow as monolayers. A 200 μl sterile pipette tip was used to scratch the cell monolayers, and the cells were then rinsed once with DMEM. Then, the cells were treated with 0.001, 0.01, 0.1, 1, and 10 μmol/L CuE, respectively, with DMEM as control. The cells were photographed, and then measured of the migration distance at 0, 24, 48, and 72 h after treatment with CuE.

### Transwell Migration Assay

After routine trypsinization of the Caco-2 cells, serum-free DMEM was used to resuspend the cells to 5 × 10^5^ cells/mL, followed by seeding the cells into the upper Transwell chambers (100 μl per well). For CuE treatment, 0.001, 0.01, 0.1, 1, and 10 μmol/L CuE diluted in serum-free DMEM (500 μl) was, respectively, added to the lower chambers, and same volume of serum-free DMEM was used as the control. Then, the cells were incubated in a 37°C incubator containing 5% CO_2_ and saturated humidity for 24 h. After the incubation, the membranes were washed twice with PBS, followed by fixing the cells on membranes with methanol for 30 min and air-drying the membranes at room temperature. The cells were stained with 0.1% crystal violet solution for 20 min, followed by washing the membranes twice with PBS, observing the membranes with miscopy to capture images from five selected fields and count the cells.

### F-Actin/G-Actin Ratio Assay

With reference to the F-actin/G-actin ratio assay described in previous study (Nakashima et al., 2010), 100 μL (1 × 10^5^ cells/mL) of Caco-2 cells were seeded in 96-well plate and incubated until 80% confluence. The cells were treated with 0.1 μmol/L CuE for 24, 48, and 72 h, with DMEM as the control. After treatment, the cells were washed thrice with PBS, and fixed with 4% paraformaldehyde (PFA) for 15 min, followed by permeabilization with 0.1% Triton for 15 min, and blocking with 2.5% BSA for 30 min. After washing three times with PBS, the cells were incubated 0.3 μM Alexa Fluor 594-phalloidin in PBS for 15 min at room temperature at the dark to label F-actin, and then incubated with 0.165 μM Alexa Fluor 488- deoxyribonuclease in PBS for 15 min at room temperature at the dark to label G-actin. After three washes with PBS, the fluorescence intensity of each well was measured with a microplate reader (Varioskan Flash, Thermo Electron Corporation, Vantaa, Finland) at 578 nm excitation wavelength, 600 nm emission, and 485 nm excitation wavelengths, 519 excitation wavelength to calculate the relative contents and the ratios of F-actin and G-actin.

### Laser Scanning Confocal Microscopy

The Caco-2 cells were seeded on collagen-coated glass coverslips and incubated until 80% confluence. The cells were treated with 0.1 μmol/L CuE for 24 h, using DMEM as the control. After treatment, the cells were fixed with 4% PFA for 15 min, permeabilized with 0.1% Triton for 15 min, and blocked with 2.5% BSA for 30 min, followed by three washes with PBS. Then, the coverslips were incubated with Alexa Fluor 594-phalloidin and 4′,6-diamidino-2-phenylindole (DAPI) for 15 min to stain F-actin and nucleus, respectively, followed by three rounds of washing with PBS. After mounted in Slowfade (Molecular Probes, Eugene, OR, United States), coverslips were imaged using a TCS SP5 laser scanning fluorescence microscopy (Leica, Germany).

### Western Blot

Western blot analysis was performed in accordance with the methods described in our previous studies (Liu et al., 2012; Cao et al., 2013), using β-actin as the loading control. The Caco-2 cells were seeded in six-well plate, and used for experiments after confluence. The cells were treated with 0.001, 0.01, 0.1, 1, and 10 μmol/L CuE for 24 h, with DMEM media as the control. Then, the cells were washed once with pre-cold PBS, followed by lysis with SDS–PAGE sample and brief sonication using a sonicator. After centrifuging at 12,000 rpm 4°C for 10 min, the supernatant was collected to boil in water bath for 5 min. Equal amounts of extracted protein from each sample was separated on SDS–PAGE, followed by transferring the proteins to PVDF membrane. The protein blot was blocked with 5% skim milk for 1 h. After incubating each individual protein blot with the corresponding primary antibodies overnight at 4°C, the membranes were washed four times in TBST (15 min each), incubated with the corresponding secondary antibodies at room temperature for 1 h, and washed another four times with TBST. Chemiluminescent reagent was used to develop the protein blots and ChemiDoc XRS system was used for chemiluminescent signal acquisition. Quantity One software (Bio-Rad Laboratories) were used for the result analysis.

### Statistical Analysis

Data are here presented as mean ± standard deviation. SPSS17.0 (SPSS Inc., Chicago, IL, United States) and Excel2013 (Microsoft, Redmond, WA, United States) software was used for statistical analysis. ANOVA was used for data analysis among groups. The *t*-test was used to compare the differences between the two groups. *P* < 0.05 was considered statistically significant. IC50 value was calculated by Excel functions. The calculating steps are as follows: (1) The LOG10 function in Excel was applied to the concentration of CuE; (2) The data of cell viability or migration were inverted into probabilities, regarding the control group as 100%; (3) The NORMSINV function was applied to regression analysis, using curve fitting to get R2 value; (4) IC50 values were then calculated through the formula of curve fitting and the POWER function.

## Results

### CuE Inhibited the Proliferation of Caco-2 Cells

**FIGURE 1:**
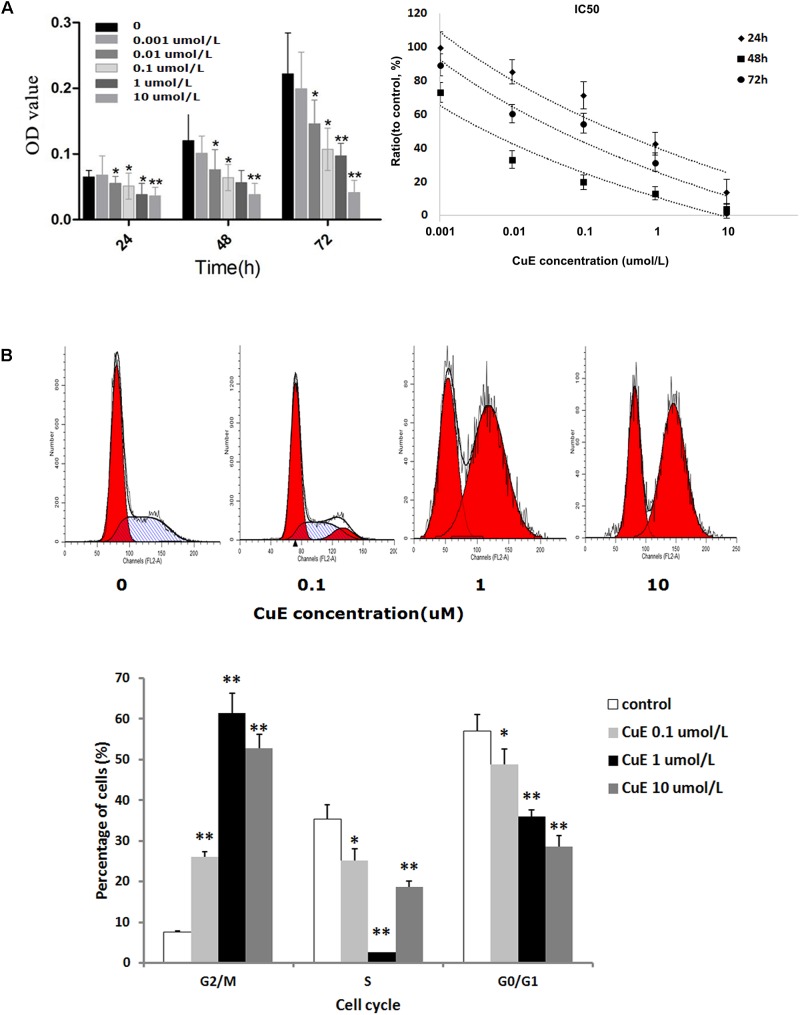
CuE-induced cofilin activation inhibited the proliferation and caused cell cycle arrest in Caco-2 cells. **(A)** Caco-2 cells were treated with CuE at the indicated dosage for 24, 48, and 72 h, respectively. The proliferation of Caco-2 cells was significantly inhibited with an IC50 ranging from 0.004 to 0.287 μM for 24, 48, and 72 h. ^∗^*P* < 0.05 and ^∗∗^*P* < 0.01, compared with the control. **(B)** Cells were treated with CuE at the indicated dosage for 24 h. The flow cytometry analysis showed cell cycle arrest at G2/M phase. Data are representative of five similar experiments.

It has been reported that the CuB-induced suppression of cell proliferation is associated with cofilin activation (dephosphorylation) (Yang et al., 2017). Thus, we investigated the effect of CuE on Caco-2 cell proliferation. As shown in **Figure 1A**, treatment of Caco-2 cells with CuE at 0.001, 0.01, 0.1, 1, and 10 μmol/L for 24, 48, and 72 h caused a dose-dependent inhibition of cell proliferation, as compared with the control. Thus, it is suggested that CuE inhibits the proliferation of intestinal epithelial cells *in vitro*.

### CuE Caused Cell Cycle Arrest in Caco-2 Cells

Previous study has demonstrated that CuB can induce G2/M phase arrest as well as formation of tetraploid cells in Jurkat cells (Zhu et al., 2012). Based on the afore-mentioned finding that the CuE inhibits the proliferation of Caco-2 cells, we further investigated the effect of CuE on cell cycle in Caco-2 cells. As illustrated in **Figure 1B**, compared with the control, treatment of Caco-2 cells with CuE for 24 h resulted in a dose-dependent reduction of both G0/G1 and S phase cells, and increase of G2/M phase cells. CuE treatment led to more cells were blocked in G2/M phase as compared with the control. It is indicated that CuE is capable of causing G2/M phase arrest in intestinal epithelial cells.

### CuE Inhibited Caco-2 Cell Migration

**FIGURE 2:**
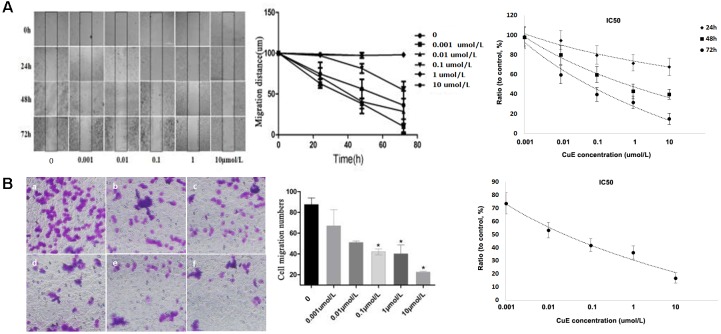
CuE-induced cofilin activation inhibited the migration of Caco-2 cells. **(A)** Caco-2 cells were treated with CuE at the indicated dosage for 24, 48, and 72 h, respectively. Cell migration was measured by the scratch assay. The migration of Caco-2 cells was inhibited in a dose- and time-dependent manner, with an IC50 ranging from 0.057 to 0.649 μM for 24, 48, and 72 h. **(B)** Cells were treated with CuE for 24 h. Transwell-based transmembrane migration assay showed the dose-dependent inhibition of transmembrane migration (IC50 = 0.022 μM). ^∗^*P* < 0.05, compared with the control. **(a)** control; **(b)** 0.001 μmol/L CuE; **(c)** 0.01 μmol/L CuE; **(d)** 0.1 μmol/L CuE; **(e)** 1 μmol/L CuE; and **(f)** 10 μmol/L CuE. Data are representative of five similar experiments.

It has been recognized that cell migration requires the activation of the underlying motility cycle, the first step of which is cell protrusion driven by actin polymerization. The early steps in actin polymerization act along with actin severing and depolymerization, which provides actin monomers for further polymerization (Ridley et al., 2003). Therefore, we investigated the effect of CuE on Caco-2 cell migration. As revealed in **Figure 2A**, the results from the scratch assay showed that treatment of Caco-2 cells with CuE for 24, 48, and 72 h inhibited cell migration in a dose- and time-dependent manner as compared with the control. In consistent with this, the results from Transwell-based transmembrane migration assay also revealed that compared with the control, CuE treatment of Caco-2 cells for 24 h caused a dose-dependent inhibition of transmembrane migration (**Figure 2B**). Thus, it is indicated that the CuE inhibits the migration of intestinal epithelial cells.

### CuE Disrupted Actin Dynamics in Caco-2 Cells

**FIGURE 3:**
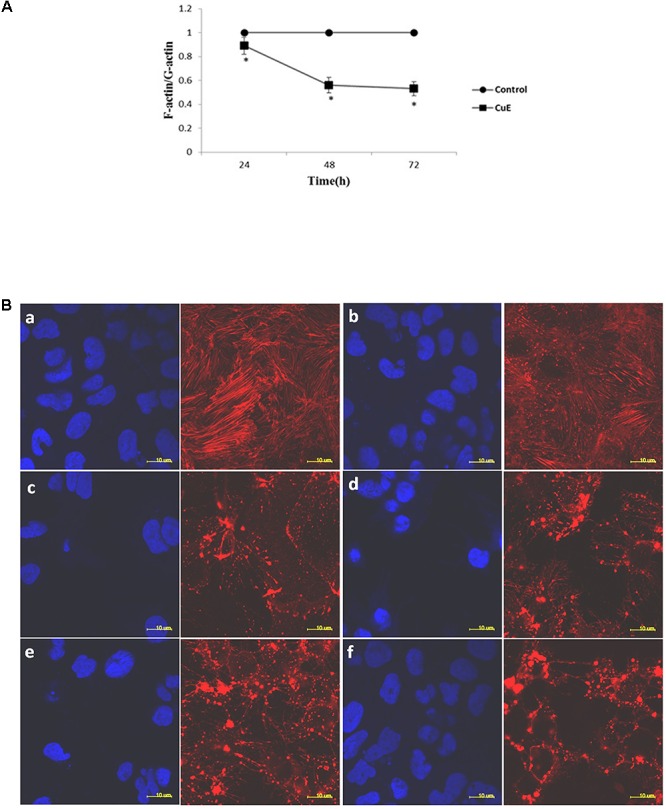
CuE-induced cofilin activation caused the disturbance of actin dynamics in Caco-2 cells. **(A)** Caco-2 cells were treated with 0.1 μmol/L CuE for 24, 48, and 72 h. The F-/G-actin ratio was significantly decreased. ^∗^*P* < 0.05, compared with the control. Data are representative of five similar experiments. **(B)** Caco-2 cells were treated with 0.1 μmol/L CuE for 1, 2, 6, 12, and 24 h, respectively. The F-actin filaments were labeled with Alexa Fluor 594-phalloidin and the nuclei were stained with DAPI. The F-actin filaments were obviously disrupted. **(a)** control; **(b)** 1 h; **(c)** 2 h; **(d)** 6 h; **(e)** 12 h; **(f)** 24 h. The red stands for F-actin and the blue represents nuclei. Scale bar = 10 μm. Data are representative of five similar experiments.

The regulation of actin dynamics is critical to numerous physical cellular processes, including cell contraction, adhesion, migration, and division. Each of these processes require precise regulation of cell shape and mechanical force generation which, to a large degree, is regulated by the dynamic mechanical behaviors of a diverse assortment of actin networks and bundles (Freischmidt et al., 2015; Salter et al., 2016; Stricker et al., 2010). Based on the above-mentioned results, we then focused on the actin dynamics in CuE-treated Caco-2 cells. In Caco-2 cells treated with 0.1 μmol/L CuE for 24, 48, and 72 h, the relative level of F-actin was similar to that of the control, whereas the relative level of G-actin was significantly higher than that of the control. The F-/G-actin ratio was significantly lower than that of the control (**Figure 3A**). Consistent with these results, confocal microscopy images revealed in **Figure 3B** also showed that the F-actin filaments were normally distributed in control Caco-2 cells. By contrast, aggregated intensive fluorescences were clearly observed in Caco-2 cells treated with 0.1 μmol/L CuE, indicating that CuE treatment caused F-actin depolymerization and aggregation, a typical appearance of F-actin cytoskeleton damage. Taken together, it is suggested that CuE is able to disrupt the actin dynamics in intestinal epithelial cells.

### CuE Induced Cofilin Activation in Caco-2 Cells

**FIGURE 4:**
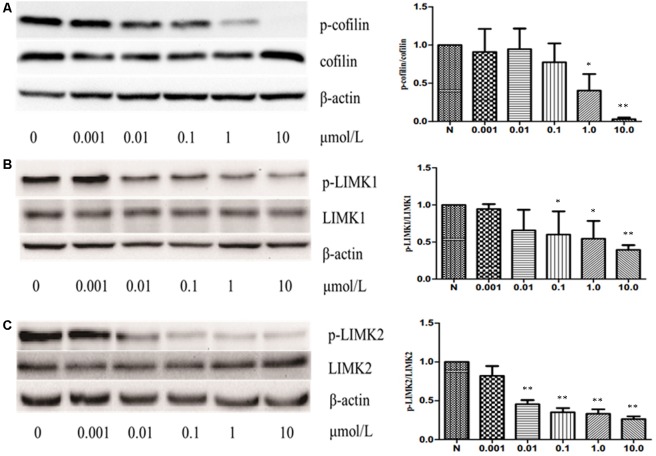
CuE induced cofilin activation in Caco-2 cells. Caco-2 cells were treated with CuE at the indicated dosage for 24 h. Cell lysates were analyzed to detect the expressions of phosphorylated cofilin **(A)**, LIMK1 **(B)** and LIMK2 **(C)** by immunoblot. CuE treatment inhibited the expressions of *p*-cofilin, *p*-LIMK1, and *p*-LIMK2 in a dose-dependent manner. Data are representative of five similar experiments. ^∗^*P* < 0.05 and ^∗∗^*P* < 0.01, compared with the control.

The previous studies have shown that some cucurbitacins such as cucurbitacin B, E, and I can activate cofilin by inhibiting the phosphorylation of cofilin in human leukemia cell lines U937 and Jurkat. Thus, to investigate whether CuE can induce cofilin activation in intestinal epithelial cells, we first detected the protein expression of total cofilin and phosphorylated cofilin in Caco-2 cells treated with CuE for 24 h. As shown in **Figure 4A**, CuE treatment did not affect the protein expression of total cofilin in Caco-2 cells. However, CuE treatment caused the protein expression of phosphorylated cofilin to decrease in a dose-dependent manner, indicating that CuE is capable of inducing cofilin activation in Caco-2 cells.

It has been well recognized that cofilin is the substrate of LIM kinase (LIMK), a kinase that plays a central role in the regulation of the actin cytoskeleton architecture by phosphorylating cofilin on serine 3 (Ser3) residue and inactivating its activity (Liu et al., 2012; Cao et al., 2013). Thus, having confirmed that CuE activates cofilin in Caco-2 cells, we next investigated whether CuE affects the cofilin upstream signaling molecule LIMK. As illustrated in **Figures 4B,C**, CuE treatment had no obvious impact on the protein expression of both LIMK1 and LIMK2 in Caco-2 cells, but inhibited the protein expression of phosphorylated LIMK1 and LIMK2 in a dose-dependent manner. These results suggest that CuE inhibits the activity of LIMK efficiently by suppressing its phosphorylation, leading to the activation of cofilin in Caco-2 cells.

## Discussion

As a natural barrier within the body, structural and functional integrity of the intestinal epithelium play an important role in facilitating normal life activities. The proliferation of intestinal epithelial cells and their migration along villi compensate for the shedding of intestinal epithelial cells, maintaining the balance and rapid regeneration of the intestinal epithelial cells. It has been well recognized that remodeling of the cytoskeleton plays a critical role during the processes of cell proliferation and migration.

Studies have shown that cucurbitacins causes marked changes in the actin cytoskeleton. Cucurbitacin is a class of tetracyclic triterpenoids extracted from the cucurbitaceae that have a wide range of pharmacological effects, such as anti-inflammatory, anti-cancer, and liver protective effects (Chen et al., 2012; Recio et al., 2012; Cai et al., 2015; Chawech et al., 2015). Among the numerous known family members of cucurbitacin, CuE is one of the more important members. The previous studies have shown that CuE significantly inhibits the proliferation and migration, and induces apoptosis in some cell types (Sörensen et al., 2012; Hsu et al., 2014). However, the effect of CuE on Caco-2 cell remains unclear. In this study, we demonstrated that CuE treatment significantly inhibits the proliferation of Caco-2 cells *in vitro*. Furthermore, we also revealed that the CuE caused G2/M phase arrest in Caco-2 cells, consequently affecting the cell proliferation. Thus, it is suggested that CuE inhibits the proliferation by inducing G2/M phase arrest in intestinal epithelial cells *in vitro*. Meanwhile, our results from both scratch assay and Transwell-based transmembrane migration assay reveal that the migration of Caco-2 cells is significantly suppressed by CuE *in vitro*. In addition, the results showed that after treatment with CuE, the F-/G-actin ratio is significantly decreased, accompanied by F-actin depolymerization and aggregation. In consistent with our findings, other investigators have demonstrated that cofilin activation increases the severing activity of cofilin, enhances the depolymerization of actin, and decreases the F-/G-actin ratio in human fibrosarcoma (HT1080) cells (Nakashima et al., 2010). Therefore, it is suggested that CuE may disrupted the actin dynamics in intestinal epithelial cells, contributing to the inhibition of cell migration *in vitro*.

Cofilin activity is closely associated with the cell proliferation and migration. It has been reported that cells lacking cofilin have cell division defects (Grintsevich et al., 2016; Wu et al., 2016). The activation of cofilin has been reported to be able to inhibit the proliferation of human myeloma cells both *in vitro* and *in vivo* (Yang et al., 2017). Similarly, it has been demonstrated that inhibition of LIMK-mediated cofilin phosphorylation could block the chemotactic migration of Jurkat cells *in vitro* (Nishita et al., 2002). On the contrary, other investigators have reported that intracellular cofilin activation promotes cell migration by generating free barbed ends, inducing protrusion, and setting the direction of cell motility (Ghosh et al., 2004). Cofilin activity is inhibited by the phosphorylation of the serine residue at position 3 (Ser-3) near the N-terminus, by the binding of phosphatidylinositol 4,5-bisphosphate [PI(4,5)P2] and cortactin, and by an increase in the intracellular pH. Cofilin activity is enhanced by actin-interacting protein-1 (AIP1) and cyclase-associated protein (CAP) (Mizuno, 2013). Some kinases such as LIMK, a serine/threonine kinase, negatively regulate the activity of cofilin by phosphorylating cofilin on Ser3 residue, thereby leading to the inactivation of cofilin; whereas some phosphatases such as slingshot dephosphorylate cofilin on Ser3 residue, resulting in the activation of cofilin (Huang et al., 2006; Li et al., 2014; Charles et al., 2015). In tumor and inflammatory cells, cofilin migrates between plasma membrane, cytosol, and actin compartments (Sparrow et al., 2012; Oleinik et al., 2014). It has been demonstrated that CuE can activate cofilin by inhibiting the phosphorylation of cofilin, leading to the increase of severing activity of cofilin and the reduction of F-/G-actin ratio in human leukemia U937 cells (Nakashima et al., 2010). In this study, we showed that CuE inhibits the phosphorylation of cofilin in intestinal epithelial cells by suppressing the phosphorylation of both LIMK1 and LIMK2, the upstream signaling kinases that regulate the dynamics of the actin cytoskeleton through the phosphorylation of cofilin. In line with our current finding, other investigators have reported that CuI, another family member of cucurbitacins, is a direct inhibitor of LIMK, as evidenced by that CuI inhibits the phosphorylation of cofilin by a direct interaction with LIMK in Hela cells (Sari-Hassoun et al., 2016).

## Conclusion

In summary, the novel finding of this study is that CuE, via activating cofilin, inhibits the cell proliferation by inducing G2/M phase arrest, and suppresses the cell migration by disrupting the actin dynamics in intestinal epithelial cells *in vitro*. It might get new insights into Cucurbitacin E mechanism of action aimed at better clarify its pharmacological effects and improve their therapeutic applications as anti-inflammatory and anti-cancer agents. Furthermore, our finding could provide an important clue of cofilin as a therapeutic target in intestinal diseases. However, the finding of this study is demonstrated only in one cell line model. To better elucidate the characterization of CuE in intestinal epithelium, it will be further explored in additional cell line models and *in-vivo* models in the following study.

## Author Contributions

HuS and YW drafted the manuscript. LL, HeS, and PW performed parts of the experiments. FW conceived the experiments and revised the manuscript.

## Conflict of Interest Statement

The authors declare that the research was conducted in the absence of any commercial or financial relationships that could be construed as a potential conflict of interest.
